# Interventions for the treatment of xerostomia: A randomized controlled clinical trial

**DOI:** 10.4317/jced.57924

**Published:** 2021-02-01

**Authors:** Constanza Marín, Laura Díaz-de-Valdés, Catalina Conejeros, René Martínez, Sven Niklander

**Affiliations:** 1 Departamento de Patología y Cirugía Oral, Facultad de Odontologia, Universidad Andres Bello, Viña del Mar, Chile

## Abstract

**Background:**

Xerostomia or dry mouth sensation corresponds to a common clinical problem that can significantly impair the oral health related quality of life (OHRQoL). Currently, there is a large variety of local agents available for the treatment of xerostomia, but there is a lack robust evidence supporting the use of one treatment over another. We aimed to compare the effectiveness of a 1% malic acid salivary stimulant spray with a 1.33% betaine-based saliva substitute mouthwash in the improvement of xerostomia.

**Material and Methods:**

Fifty-one participants with drug induced or idiopathic xerostomia were randomly allocated into three groups, two intervention and one control group (placebo). OHRQoL and severity of xerostomia were assessed with the OHIP-14sp questionnaire and a visual analogue scale (VAS) respectively. Stimulated and non-stimulated salivary flow rates before and after treatments were also measured.

**Results:**

All three groups reported a significantly improvement in the dry mouth sensation and non-stimulated salivary flow rates, but only the malic acid spray and the betaine-based mouthwash significantly improved the OHRQoL. There were no significant differences between both intervention groups.

**Conclusions:**

This study provides evidence that both tested agents are comparable in improving the dry mouth sensation and OHRQoL of patients with drug induced and idiopathic xerostomia.

** Key words:**Xerostomia, dry mouth, malic acid, betaine, oral-health related quality of life.

## Introduction

The term xerostomia corresponds to a clinical symptom defined as a “subjective impression of oral dryness” (1,2). This is term is based on an individual’s own sensation of dry mouth, which depending on the cause is not necessarily correlated with an objective decrease in the amount of saliva (hyposalivation) (3). Xerostomia with or without hyposalivation can impair speaking (4,5), chewing (4,5), tasting (6) and swallowing (1,3), having a significant impact on the oral health-related quality of life (OHRQoL) (7). When xerostomia is accompanied with hyposalivation, individuals are at a higher risk of developing dental caries (2,4), gingivitis (4), erosion and ulceration of mucosal tissues (6), oral candidiasis, dysgeusia and dysphagia (6,8), which have also a detrimental effect on the OHRQoL (7,9).

Xerostomia affects millions of persons worldwide, with an estimated prevalence that ranges from 10-46% (1,5). Dry mouth sensation is most common in menopausal women and in individuals above 65 years of age (10). Medication is considered as the main cause of xerostomia (6,11). In fact, more than 400 medications are associated with xerostomia; antidepressants, antihypertensives, and antihistamines being the most commons (6). It is not only the type of medication that matters, but also the number of drugs being taken. In a previous report we estimated that the chance of having xerostomia increases 1.12 times with each additional medication (7). Other common causes of xerostomia include head and neck radiotherapy, Sjögren’s syndrome, anxiety and depression, systemic sclerosis, sarcoidosis, Parkinson’s syndrome, among others (2,5,10).

Appropriate patient assessment, including a comprehensive medical-dental history and diagnostic tests, e.g. salivary flow measurement, are essential when diagnosing xerostomia (12). A decrease in the amount of saliva accompanying the dry mouth sensation requires further investigation, as this might suggest a systemic condition affecting the salivary glands (e.g. Sjögren’s syndrome).

In order to treat xerostomia, it is important to identify the underlying cause, as xerostomia is a symptom, not a disease itself. If xerostomia is being caused by a systemic condition, it is important to have that condition under medical control, although that will not necessarily improve the dry mouth sensation by itself. If xerostomia is secondary to medication intake, an attempt with the patient’s physician could be done to replace that medication (6), although that is not always possible. Also, xerostomia is sometimes caused by a combination of drugs, rather than by a single drug itself (7).

Currently, there are plenty of available options for the treatment of xerostomia, which include systemic and local therapies. In patients in which xerostomia is being caused by hyposalivation (with remaining glandular acini), systemic therapies such as cevimeline or pilocarpine might be of used (4,13), although they can have significant adverse side-effects (13). Local therapies, which include salivary stimulants and salivary substitutes, are the most preferred treatment options, as they produce symptom relieve with little or no adverse side effects. Nevertheless, there is a lack of substantial evidence supporting the use of one treatment above another for the relieve of dry mouth sensation (14). We previously reported that a topical salivary stimulant containing 1% malic acid (Xeros Dentaid Spray) was effective in improving dry mouth sensation and OHRQoL of patients with drug induced or idiopathic xerostomia (15). In the present study, we aimed to compare the effect of a 1% malic acid spray (Xeros Dentaid Spray) with a 1,33% betaine- 0,10% allantoin saliva substitute mouthwash (Xeros Dentaid Mouthwash) in the improvement of dry mouth sensation and OHRQoL of patients with xerostomia, in order to provide evidence of which of this agents is superior in the treatment of xerostomia.

## Material and Methods

-Patients and study design

Only patients over 18 years of age were enrolled in this study. Patients who had history of head and neck radiotherapy, chemotherapy, and/or any systemic disease reported to produce hyposalivation (Sjögren’s syndrome, scleroderma, hepatitis C, HIV, sarcoidosis, rheumatoid arthritis, polyarteritis nodosa, systemic sclerosis, or lupus erythematosus) were excluded from this study. The study involved 51 patients with xerostomia who attended to the Dentistry Faculty of Andres Bello University, Viña del Mar- Chile, between April to September of 2016. Written informed consent was obtained from all patients, who voluntarily agreed to participate in this study. The Ethical and Scientific Committee of the Dentistry Faculty of Andres Bello University approved this study (approval number 43-2016). This research was conducted in full accordance with the World Medical Association Declaration of Helsinki. The study was designed as randomized clinical trial following guidelines established by The Consort Statement (http://www.consort-statement.org/consort-statement/).

Patients with xerostomia were randomly distributed into three groups of 17 individuals each (two intervention groups and one control group). Randomization was done by an investigator not involved in the study through a specific web page (http://www.randomization.com/) using the method of randomly permuted blocks.

-Sample size calculation

Sample size was calculated using G Power 3.0.10. We set the significance level and power of the study at 5% and 95% respectively. Proportions were obtained from our previous paper (15). Calculated sample size was of 17 participants for each group.

-Interventions

**Figure 1:**
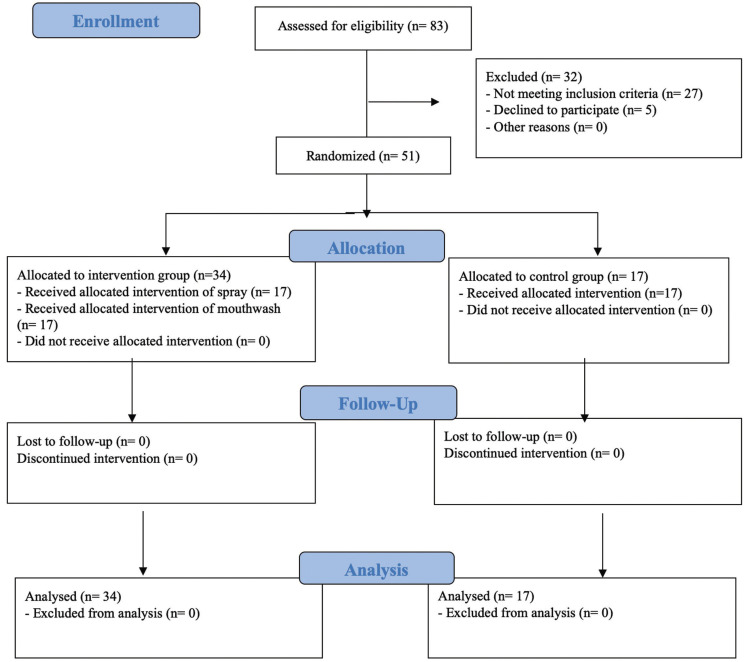
Study CONSORT flow chart.

After randomization, 51 patients were distributed into three groups of 17 individuals each (two intervention and one control group) (Fig. 1). In one of the interventions groups, patients received a topical salivary stimulant spray comprising 1% malic acid, 10% xylitol, and 0.05% sodium fluoride (Xeros Dentaid Spray; Dentaid) while in the other intervention group patients received a saliva substitute mouthwash comprising 1,33% betaine, 0,10% allantoin, 3,30% xylitol and 0.05% sodium fluoride (Xeros Dentaid Mouthwash; Dentaid). In the control group, patients received a placebo spray comprising 10% xylitol and 0.05% sodium fluoride. Each formulation was placed into identical opaque flasks (with atomizer) and labelled according to randomization by personnel unrelated to the study. The atomizer of the mouthwash preparation was blocked, so in case of receiving a non-working atomizer, patients were instructed to open the bottle and use it as a mouthwash, without telling the researchers. All participants were instructed to use the allocated treatment for a period of 2 weeks, no more than 8 times a day. We had no losses during the study and no adverse side effects were reported. To assess compliance, patients had to write in a diary the number of applications per day.

-Measurements

•Xerostomia

Xerostomia was assessed as follows. Participants were asked the following question as reported in previews articles (15,16): “How often do you feel that your mouth is dry?”. Participants could select from the following answers: “never”, “sometimes”, “usually”, or “always”. Those who answered “usually” or “always” were considered to have xerostomia and were asked to mark in a visual analogue scale (VAS) how dry their mouth was. Of those participants, the ones that scored 30 mm or higher in the VAS were invited to participate in the study.

•Severity of xerostomia

The severity of xerostomia was assessed using the visual analogue scale (VAS), which comprised a 100 mm horizontal line with a “0” and “100” marked on each extreme. A score of 0 indicated “no xerostomia” and 100 indicated the “worst imaginable xerostomia”. All patients were asked to draw a vertical line perpendicular to this horizontal line to reflect their symptom severity. We evaluated and recorded the distance between the vertical line and the zero extreme to obtain the VAS score for each patient (8,9). Only patients scoring 30 mm or more were enrolled.

•Evaluation of impact on the quality of life 

The OHRQoL was assessed using the Spanish version of the Oral Health Impact Profile-14 questionnaire (OHIP-14sp) before and after the treatment (19). OHIP-14 is a 14-item questionnaire designed to assess self-reported functional limitation, discomfort, and disability attributed to oral conditions. The OHIP-14sp has been proven to be reliable, sensitive to changes, and have adequate cross-cultural consistency (19,20). We evaluated the OHIP-14sp according to the following dimensions: functional limitation, physical pain, psychological discomfort, physical incapacity, psychological incapacity, social incapacity, and social disadvantage. The answers were assessed using a Likert type evaluation scale with five points as follows: never = 0; rarely = 1; sometimes = 2; repeatedly = 3; and always = 4. Of note, the OHIP-14sp scale ranges from 0 to 56 (20). The lowest scores represent a satisfactory perception of an individual’s oral conditions and, therefore, a higher satisfaction and better quality of life.

•Salivary flow rate

We assessed stimulated and non-stimulated salivary flow rates before and after treatment using the spitting method. All patients were instructed to refrain from eating, drinking, smoking, and oral hygiene procedures for a minimum of 60 min before the procedure. Samples were collected in the morning hours, between 9:30 and 11:30 am, always in the same room under similar room temperatures. The collection time for stimulated and non-stimulated whole salivary flow was 5 min. First, non-stimulated whole saliva was collected. Patients were instructed to spit into a previously weighted cup for 5 min. The cup was then weighted using a precision balance. The salivary flow rate was calculated in ml/min using the following formula: X=p2-p1/T/1.005, where p1 is the initial weight of the cup, p2 the weight of the cup with saliva, T the time of collection, and 1.005 the saliva estimated weight (g/ml) (21).

After a break of three minutes, stimulated whole saliva was collected using the mastication method. Patients were asked to chew a wax cube of 15 × 10 mm for 1 min at their own pace and then to spit into a cup for 5 min. Wax residues were eliminated using a filter paper. Quantification was done using the method previously described.

-Outcomes

•Impact on OHRQoL: The impact of a 1% malic acid salivary stimulant spray and a 1,33% betaine-based saliva substitute mouthwash on the OHRQoL was defined as the difference between the baseline total OHIP-14sp scores and post-treatment total OHIP-14sp scores (19,20).

•Dry mouth symptoms: Measured through a visual analogue scale (VAS) before and after intervention (17,18).

•Salivary flow stimulation: Defined as the difference between the stimulated and non-stimulated salivary flows before and after treatment, expressed as mL/min.

Both primary and secondary outcomes were measured 2 days after patients finished the 2-week treatment, whether with the placebo, saliva substitute or saliva stimulant (15).

-Statistical Analysis

Data were analysed using Graphpad Prism 7 Software. Shapiro-Wilk test was applied to study the normality of all groups. When related samples were not normally distributed, the Wilcoxon test was performed. In all other cases, the t-Student test was applied. The comparison between interventions was studied using ANOVA or Kruskal-Wallis tests, according to whether the variables had a normal distribution or not, respectively. Values ≤ 0.05 were considered as statistically significant.

## Results

Fifty-one patients were randomly allocated to any of the three study groups; 17 to the 1% malic acid spray group (Xeros Dentaid Spray), 17 to the 1,33% betaine-based mouthwash group (Xeros Dentaid Mouthwash) and 17 to the control group (a placebo containing 10% xylitol and 0.05% sodium fluoride). Groups were matched in terms of age and gender (Table 1). Xerostomia was associated to drug use in 46 patients (90%), while in the other 5 patients (10%) xerostomia was associated to idiopathic causes. No differences were observed between groups (Table 1). Hyposalivation (defined as a decrease in the stimulated salivary flow) (2) was observed in 10 patients (19.6%); 4 from the malic acid group, 4 from the control group and 2 from the betaine group. No differences in the initial VAS and OHIP-14 scores, non-stimulated and stimulated salivary flow rates and the mean number of medications were observed between groups (*p* > 0.05) (Table 1).

**Table 1:**
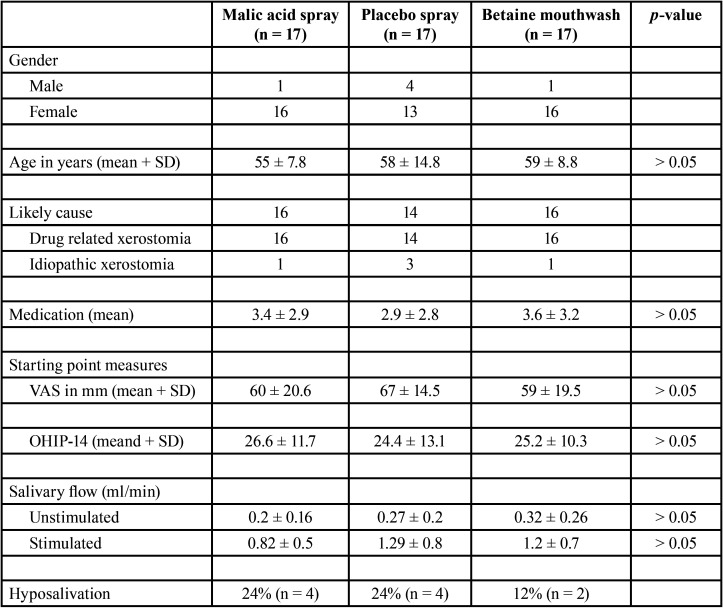
Demographics and initial OHIP-14, VAS and salivary flow values of each study group.

**Figure 2:**
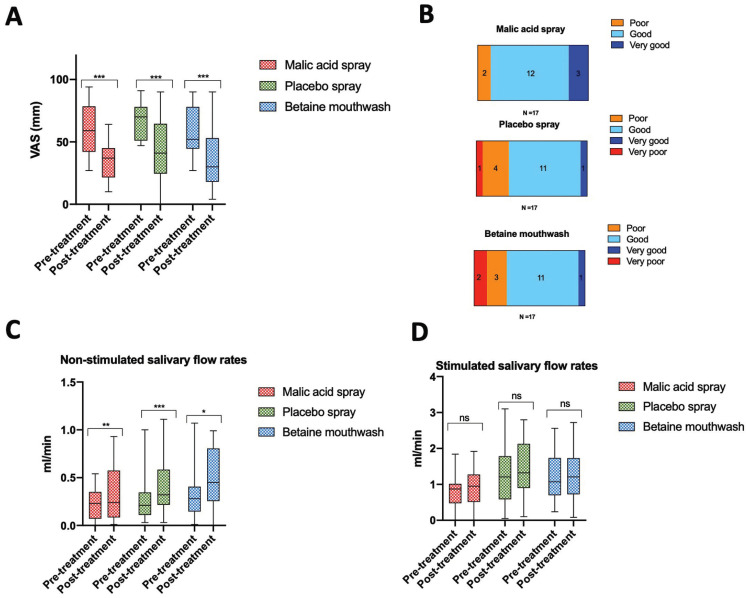
Patients self-perception of dry mouth and salivary flow rates before and after treatment. Both interventions and the placebo significantly reduced dry mouth sensation after two weeks of treatment. No differences between the three groups were observed (A). Most of the patients (in each of the groups) felt the intervention they were receiving was having a good or very good effect. No differences between the three groups were observed (B). All three groups experienced a significant increase in non-stimulated salivary flow rates after two weeks of treatment, with no significant differences between groups (C). No significant differences in stimulated salivary flow rates were observed in any of the groups (D). * *p* < 0.05, ** *p* < 0.005, *** *p* < 0.0005.

After the intervention period, all three groups (malic acid, control and betaine) reported to have a statistically significant improvement in the severity of xerostomia, measured by the VAS (*p* = 0.0001 for the malic acid group, *p* = 0.0001 for the control group and *p* = 0.0004 for the betaine group). No differences were observed between the groups (*p* > 0.05) (Fig. 2A). When asked, “how effective the treatment was in reducing your dry mouth sensation”, most patients answered good or very good (15 in the malic acid,12 in the control group and 12 in the betaine mouthwash group). No differences were observed between groups (*p* > 0.05) (Fig. 2B).

All three interventions (malic acid spray, placebo spray and the betaine mouthwash) significantly increased non-stimulated salivary flow rates (*p* = 0.0039 for the malic acid group, *p* = 0.0005 for the control group and *p* = 0.03 for the betaine group) (Fig. 2C) but not stimulated salivary flow rates (*p* > 0.05) (Fig. 2D). No differences between groups were observed, neither for non-stimulated nor stimulated salivary flow rates (*p* >0.05).

**Figure 3:**
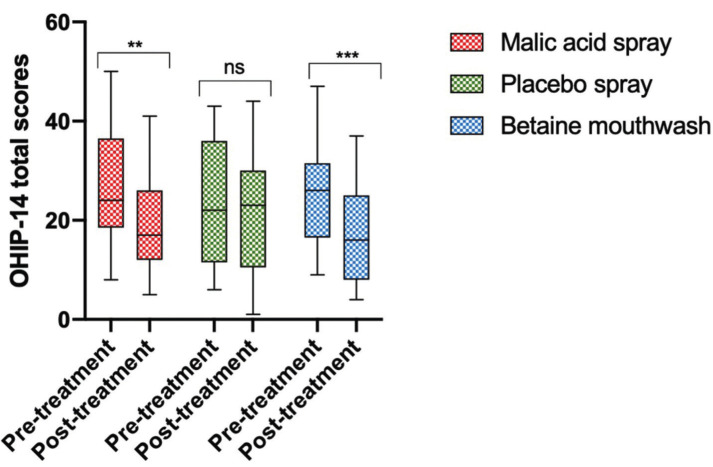
Both the malic acid spray and the betaine-based mouthwash significantly improved the OHRQoL. Both interventions (malic acid spray and betaine mouthwash) but not the placebo, significantly improved the OHRQoL, as shown by a significant decrease in the total OHIP-14 scores. No significant differences were observed between both interventions. ** *p* < 0.005, *** *p* < 0.0005 .

After two weeks of treatment, both the malic acid spray and the betaine mouthwash significantly improved the OHRQoL (*p* = 0.0019 for the malic acid spray and *p* = 0.0006 for the betaine mouthwash). No differences were observed between both intervention groups (*p* > 0.05). No improvement was observed in the placebo group (*p* > 0.05) (Fig. 3). Patients from the betaine mouthwash group had a significant improvement in six of the seven dimensions of the OHIP-14 questionnaire (all apart from social disability), whereas individuals from the malic acid group had a significant improvement in four dimensions (physical pain, physical disability, psychological disability, handicap). Individuals from the control group had a significant increase in only one dimension (psychological disability) (Table 2).

**Table 2:**
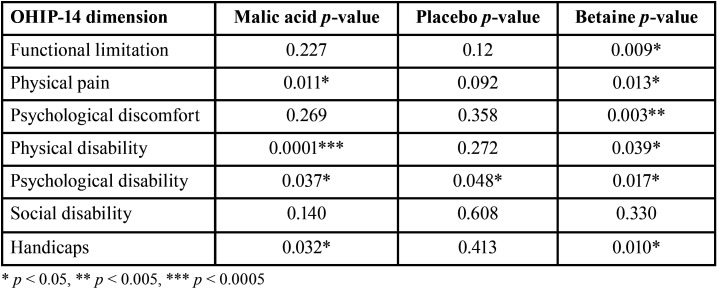
Improvement of the different OHIP-14 dimensions in each study group.

## Discussion

Xerostomia corresponds to a very common clinical problem with an increasing prevalence (5), probably because of an increase in medication intake due to age-related pathologies (4). Xerostomia with or without hyposalivation can significantly affect the OHRQoL (7,22–24), thus it is important to find treatment agents that can improve the dry mouth sensation, but more importantly, improve the OHRQoL of the affected individuals. Currently, there is a lack of robust evidence supporting one treatment over another (14,25), so the selection of treatment agents is basically done based on personal preferences. We previously reported that a salivary stimulant spray containing 1% malic acid (Xeros Dentaid Spray) was successful in improving dry mouth sensation and OHRQoL of patients with xerostomia when compared to a placebo (15). In this study, we aimed to compare the effectiveness of a saliva substitute containing 1.33% betaine and 0.10% allantoin (Xeros Dentaid Mouthwash) with a salivary stimulant spray containing 1% malic acid (Xeros Dentaid Spray) in improving the OHRQoL and dry mouth sensation of patients with drug-induced or idiopathic xerostomia, in order to provide evidence of which of these agents is superior in the treatment of xerostomia.

Drug intake is the most common cause of dry mouth (6,11). In the present study, we only included patients with drug-induced or idiopathic xerostomia (90% of the patients were taken medications for chronic conditions), as it is known that in both, the salivary glands have no irreversible damage. It is thought that both forms of xerostomia (idiopathic and drug related) are caused by the affection of salivary gland innervation, at either the central or peripheral level (26). This means that in theory, affected patients could benefit of both, salivary stimulants and salivary substitutes.

We observed that the three study groups (malic acid, control and betaine groups) had a statistically significant improvement in the dry mouth sensation measured by the VAS. None of the tested interventions was superior than the other. It was not a surprise that the control group had also a decrease in the dry mouth sensation, as in our previous study (15) we found similar results. This can be explained by the fact that patients in the control group used a placebo spray consisting in 10% xylitol and 0.05% sodium fluoride, and xylitol can act as an active placebo increasing the salivary flow rate (27). In fact, it seems that this was the case in the present study, as a significant increase in non-stimulated salivary flow rates were observed in all three groups, including the control group. Our results are aligned with the ones reported by Gómez-Moreno *et al.* (28) and Ship *et al.* (29) who reported a decrease in dry mouth sensation after treatment with 1% malic acid and a betaine-based mouthwash, respectively.

As mentioned above, a significant increase in non-stimulated salivary flow rates were observed in all three groups. No significant increase in stimulated salivary flow rates were observed in any of the groups. An explanation for this might be that most of the patients included in this study had xerostomia without hyposalivation. Also, no patients with known conditions to produce irreversible damage to the salivary glands were included, thus the salivary glands were fully functional (2,11). Thus, under proper stimulation (e.g. mastication), the salivary glands were able to respond as expected, increasing the salivary output. Hence, any effect induced by the intervention had to be very significant in order to reach statistical significance. In fact, in all three groups, a minor increase in stimulated salivary flow rate was observed, but in none of them reached statistical significance. Because the salivary output is lower under no stimulation, the improvement induced by the interventions in non-stimulated salivary flow rates was more noticeable, thus reached statistical significance.

These results are similar to the ones reported from previous reports (15,27,28) where a 1% malic acid spray was shown to significantly increase unstimulated and stimulated salivary flow rates after two weeks of use. Similar to this, betaine containing mouthwashes (29,30) have also shown to increase non-stimulated salivary flow rates, although this is likely to be due to the xylitol present in betaine-based mouthwashes rather than from the betaine itself (29).

Both interventions, the 1% malic acid spray (Xeros Dentaid Spray) and the 1.33% betaine-based mouthwash (Xeros Dentaid Mouthwash), but not the placebo, were successful in improving the OHRQoL of patients with xerostomia. Individuals using the betaine-based mouthwash had a significant improvement in 6 of the 7 dimensions of the OHIP-14 questionnaire, whereas individuals using the malic acid spray had an improvement in 4. Nevertheless, none of the interventions showed to be superior than the other in improving the overall OHRQoL, as no statistical differences were observed between both groups. Both interventions have been reported previously to increase the OHRQoL of patients with xerostomia (15,27–30), but it is difficult to compare our results with other published reports, since until now, no clinical trials have compared both interventions between each other.

This study had some limitations. Although we included a control group where patients had to use a placebo spray, it would have been ideal to have a second control group with a mouthwash placebo. This would have allowed to do a more reliable comparison between the control group and the betaine mouthwash group. Nevertheless, the main aim of this study was to compare both interventions between each other, rather than compared them with a control group. Also, we just included patients with drug induced or idiopathic xerostomia, where patients are not expected to have glandular damage, so these results are not transposable to other causes of xerostomia in which glandular damage is common (e.g. Sjögren’s syndrome, post head and neck radiotherapy, among others)

Currently, there are no standardized protocols for the treatment of dry mouth. There are many reports showing a variety of treatment agents to have different success rates in the relieve of xerostomia, but there are not many clinical trials comparing two or more interventions in terms of OHRQoL improvement. Thus, in clinical settings, it is very difficult to choose a single treatment when prescribing to patients, as there is a lack of evidence supporting one treatment over another. In this study, we provided evidence that both interventions, the 1% malic acid salivary stimulant spray (Xeros Dentaid Spray) and the 1.33% betaine-based saliva substitute mouthwash (Xeros Dentaid Mouthwash), are equally able to improve the OHRQoL and dry mouth sensation of patients with drug-induced or idiopathic xerostomia.

## References

[1] Villa A, Abati S (2011). Risk factors and symptoms associated with xerostomia: a cross-sectional study. Aust Dent J.

[2] Ying Joanna ND, Thomson WM (2015). Dry mouth - An overview. Singapore Dent J.

[3] Ikebe K, Matsuda K, Morii K, Wada M, Hazeyama T, Nokubi T (2007). Impact of dry mouth and hyposalivation on oral health-related quality of life of elderly Japanese. Oral Surg Oral Med Oral Pathol Oral Radiol Endod.

[4] Gil-Montoya JA, Silvestre FJ, Barrios R, Silvestre-Rangil J (2016). Treatment of xerostomia and hyposalivation in the elderly: A systematic review. Med Oral Patol Oral Cir Bucal.

[5] Hopcraft MS, Tan C (2010). Xerostomia: an update for clinicians. Aust Dent J.

[6] Yuan A, Woo SB (2015). Adverse drug events in the oral cavity. Oral Surg Oral Med Oral Pathol Oral Radiol.

[7] Niklander S, Veas L, Barrera C, Fuentes F, Chiappini G, Marshall M (2017). Risk factors, hyposalivation and impact of xerostomia on oral health-related quality of life. Braz Oral Res.

[8] Ohara Y, Hirano H, Yoshida H, Obuchi S, Ihara K, Fujiwara Y (2016). Prevalence and factors associated with xerostomia and hyposalivation among community-dwelling older people in Japan. Gerodontology.

[9] van der Putten GJ, Brand HS, Schols JM, de Baat C (2011). The diagnostic suitability of a xerostomia questionnaire and the association between xerostomia, hyposalivation and medication use in a group of nursing home residents. Clin Oral Investig.

[10] Tanasiewicz M, Hildebrandt T, Obersztyn I (2016). Xerostomia of Various Etiologies: A Review of the Literature. Adv Clin Exp Med.

[11] Scully C, Bagan JV (2004). Adverse drug reactions in the orofacial region. Crit Rev Oral Biol Med.

[12] Plemons JM, Al-Hashimi I, Marek CL, American Dental Association Council on Scientific Affairs (2014). Managing xerostomia and salivary gland hypofunction: executive summary of a report from the American Dental Association Council on Scientific Affairs. J Am Dent Assoc.

[13] Villa A, Connell CL, Abati S (2014). Diagnosis and management of xerostomia and hyposalivation. Ther Clin Risk Manag.

[14] Furness S, Worthington HV, Bryan G, Birchenough S, McMillan R (2011). Interventions for the management of dry mouth: topical therapies. Cochrane Database Syst Rev.

[15] Niklander S, Fuentes F, Sanchez D, Araya V, Chiappini G, Martinez R (2018). Impact of 1% malic acid spray on the oral health-related quality of life of patients with xerostomia. J Oral Sci.

[16] Murray Thomson W, Poulton R, Mark Broadbent J, Al-Kubaisy S (2006). Xerostomia and medications among 32-year-olds. Acta Odontol Scand.

[17] Minicucci EM, Pires RB, Vieira RA, Miot HA, Sposto MR (2013). Assessing the impact of menopause on salivary flow and xerostomia. Aust Dent J.

[18] Pai S, Ghezzi EM, Ship JA (2001). Development of a Visual Analogue Scale questionnaire for subjective assessment of salivary dysfunction. Oral Surg Oral Med Oral Pathol Oral Radiol Endod.

[19] Lopez R, Baelum V (2006). Spanish version of the Oral Health Impact Profile (OHIP-Sp). BMC Oral Health.

[20] Montero-Martín J, Bravo-Pérez M, Albaladejo-Martínez A, Hernández-Martín LA, Rosel-Gallardo EM (2009). Validation the Oral Health Impact Profile (OHIP-14sp) for adults in Spain. Med Oral Patol Oral Cir Bucal.

[21] Navazesh M, Kumar SK (2008). University of Southern California School of Dentistry. Measuring salivary flow: challenges and opportunities. J Am Dent Assoc.

[22] Thomson WM, Lawrence HP, Broadbent JM, Poulton R (2006). The impact of xerostomia on oral-health-related quality of life among younger adults. Health Qual Life Outcomes.

[23] Thomson WM (2015). Dry mouth and older people. Aust Dent J.

[24] Ahmad MS, Bhayat A, Zafar MS, Al-Samadani KH (2017). The Impact of Hyposalivation on Quality of Life (QoL) and Oral Health in the Aging Population of Al Madinah Al Munawarrah. Int J Environ Res Public Health.

[25] Furness S, Bryan G, McMillan R, Birchenough S, Worthington HV (2013). Interventions for the management of dry mouth: non-pharmacological interventions. Cochrane Database Syst Rev.

[26] Silvestre-Donat FJ, Miralles-Jordá L, Martínez-Mihi V (2004). Protocol for the clinical management of dry mouth. Med Oral.

[27] Gómez-Moreno G, Aguilar-Salvatierra A, Guardia J, Uribe-Marioni A, Cabrera-Ayala M, Delgado-Ruiz RA (2013). The efficacy of a topical sialogogue spray containing 1% malic acid in patients with antidepressant-induced dry mouth: a double-blind, randomized clinical trial. Depress Anxiety.

[28] Gómez-Moreno G, Guardia J, Aguilar-Salvatierra A, Cabrera-Ayala M, Maté-Sánchez de-Val JE, Calvo-Guirado JL (2013). Effectiveness of malic acid 1% in patients with xerostomia induced by antihypertensive drugs. Med Oral Patol Oral Cir Bucal.

[29] Ship JA, McCutcheon JA, Spivakovsky S, Kerr AR (2007). Safety and effectiveness of topical dry mouth products containing olive oil, betaine, and xylitol in reducing xerostomia for polypharmacy-induced dry mouth. J Oral Rehabil.

[30] Martín M, Marín A, López M, Liñán O, Alvarenga F, Büchser D (2017). Products based on olive oil, betaine, and xylitol in the post-radiotherapy xerostomia. Rep Pract Oncol Radiother.

